# Hashimoto’s encephalopathy in psychiatric inpatients: neuropsychiatric morbidity, diagnostic challenges and treatment

**DOI:** 10.3389/fpsyt.2025.1639179

**Published:** 2025-09-10

**Authors:** Rifat Serav İlhan, Kazım Cihan Can, Şafak Yalçın Şahiner, Berker Duman, Burçin Çolak, Yağmur Kır, Seyda Erdoğan, Rezzak Yilmaz, Canan Yücesan, Mine Araz, Gule Çınar, Sena Ünal, Özlem Doğan, Emine Uslu, Meram Can Saka

**Affiliations:** ^1^ Department of Psychiatry, Ankara University School of Medicine, Ankara, Türkiye; ^2^ Department of Neurology, Ankara University School of Medicine, Ankara, Türkiye; ^3^ Department of Nuclear Medicine, Ankara University School of Medicine, Ankara, Türkiye; ^4^ Department of Clinical Microbiology and Infectious Diseases, Ankara University School of Medicine, Ankara, Türkiye; ^5^ Department of Radiology, Ankara University School of Medicine, Ankara, Türkiye; ^6^ Department of Medical Biochemistry, Ankara University School of Medicine, Ankara, Türkiye; ^7^ Department of Rheumatology, Ankara University School of Medicine, Ankara, Türkiye

**Keywords:** Hashimoto’s encephalopathy, autoimmune encephalitis, autoimmune psychosis, catatonia, steroid-responsive encephalopathy associated with autoimmune thyroiditis (SREAT)

## Abstract

**Introduction:**

Hashimoto’s encephalopathy (HE) is a rare neuro-inflammatory disorder that poses a significant diagnostic challenge, particularly when its clinical presentation is dominated by psychiatric symptomatology. This study aimed to delineate the constellation of clinical and paraclinical features that differentiate HE from primary psychiatric disorders among seropositive inpatients, thereby facilitating its early and accurate identification.

**Methods:**

In this retrospective, single-center study, the records of 484 consecutively admitted female psychiatric inpatients were reviewed. The final cohort comprised 40 patients with confirmed thyroid autoantibody seropositivity. Patients presenting with atypical features or suspected autoimmune etiologies were subjected to a comprehensive neurodiagnostic evaluation, including cerebrospinal fluid (CSF) analysis, electroencephalography (EEG), MRI, and 18F-fluorodeoxyglucose positron emission tomography (FDG-PET). The diagnosis of HE was established based on stringent criteria, including the exclusion of alternative etiologies and a definitive therapeutic response to corticosteroids.

**Results:**

Of the 40 seropositive patients, nine (22.5%) met the diagnostic criteria for definitive HE. The HE cohort exhibited a significantly later age of psychiatric symptom onset (42.9 *vs*. 30.1 years; *p*=0.011) and markedly greater functional impairment (GAF score: 31.7 *vs*. 51.6; *p*<0.001) compared to the non-HE group. The most discriminating clinical features were delirium (88.9% in HE *vs*. 6.5% in non-HE; *p*<0.001) and catatonia (77.8% *vs*. 32.3%; *p*=0.015). Corroborating neurodiagnostic evidence for HE included inflammatory CSF abnormalities (55.6% *vs*. 0%), diffuse EEG slowing (33.3% *vs*. 0%), and cortical hypometabolism on FDG-PET (85.7% *vs*. 0%). All patients with HE were refractory to standard psychiatric interventions but achieved prompt and substantial clinical remission following high-dose corticosteroid administration.

**Discussion:**

In conclusion, HE manifests as a distinct and severe neuropsychiatric syndrome in a subset of seropositive psychiatric inpatients. The emergence of a late-onset clinical picture characterized by a delirium-catatonia complex, particularly when refractory to conventional psychiatric treatment, warrants a high index of suspicion for HE. A multimodal diagnostic approach is essential for accurate identification, enabling the timely initiation of immunotherapy to ameliorate severe neuropsychiatric morbidity and prevent long-term disability

## Introduction

### Background

Hashimoto’s Encephalopathy (HE), first identified in 1966, is a rare neurological condition associated with thyroid autoimmunity (1). Also known as steroid-responsive encephalopathy associated with autoimmune thyroiditis (SREAT), it presents a broad spectrum of symptoms, including cognitive impairment, seizures, stroke-like episodes, psychiatric disturbances, and altered consciousness (2, 3). While linked to high levels of thyroid autoantibodies like anti-thyroid peroxidase (anti-TPO) and anti-thyroglobulin (anti-TG), the exact role of these antibodies in its pathogenesis remains unclear (2).

HE’s clinical presentation is highly variable, with psychiatric symptoms often dominating early stages. Symptoms such as psychosis, depression, catatonia, and mood disorders can lead to confusion with primary psychiatric disorders, increasing the risk of misdiagnosis (4, 5). This is particularly problematic in psychiatric settings, where HE may be overlooked, delaying treatment and worsening patient outcomes. For example, psychiatric patients are often treated with antipsychotics, which may be ineffective for HE, necessitating immunotherapies like corticosteroids instead (4).

With a prevalence estimated at 2.1 per 100,000, HE is four times more common in women, with an average onset age of 40–55 years (6). Despite its rarity, early diagnosis is critical, as corticosteroid treatment can reverse symptoms and improve outcomes (3). Delayed diagnosis, however, can lead to permanent neurological deficits or unnecessary psychotropic medication use, posing significant management challenges.

Most research has focused on neurological cohorts, where seizures, myoclonus, and stroke-like episodes are commonly reported (7). Yet, data on HE in psychiatric populations are limited, and its recognition is challenging when psychiatric symptoms predominate, highlighting a gap in psychiatric practice awareness (5). Addressing this gap is essential to optimize diagnostic accuracy and therapeutic outcomes in psychiatric inpatients presenting with atypical, treatment-resistant symptom profiles.

### Rationale and objectives

In an exploratory, single-center cohort of consecutively admitted female psychiatric in-patients subjected to routine thyroid-antibody screening and comprehensive paraclinical work-up, the present study objectives sought to:

1. Generate preliminary estimates for the proportion of confirmed HE among seropositive admissions in psychiatric inpatients
2. Delineate the constellation of clinical, cognitive, and neurodiagnostic parameters that best distinguish HE from alternative psychiatric or neurological diagnoses; and

By identifying salient differentiating features, this study also aims to inform the design of prospective trials and to facilitate earlier, evidence-based recognition of HE in psychiatric practice.

These objectives are designed to improve recognition and management of HE in psychiatric contexts, particularly in treatment-resistant cases, reducing morbidity through early intervention. By focusing on psychiatric inpatients, the study addresses a critical gap, potentially informing diagnostic protocols and treatment guidelines.

## Methods

### Study design and setting

This study was a retrospective chart review conducted at a tertiary psychiatric inpatient facility in Ankara, Turkey, within a specialized women’s neuropsychiatry unit. This unit functions as a referral center for complex cases characterized by severe psychiatric symptoms, systematically evaluating patients for both primary psychiatric disorders and underlying organic etiologies, including neurodevelopmental, neurodegenerative, and neuroimmunological conditions. The clinical approach prioritizes comprehensive differential diagnosis, employing paraclinical investigations to identify potentially reversible or treatable medical contributors to psychiatric syndromes.

Since 2022, all inpatients have undergone standardized clinical and paraclinical assessments within a real-world clinical framework, emphasizing the systematic identification of organic contributors to psychiatric symptomatology. Patients with suspected autoimmune neuropsychiatric conditions, including potential Hashimoto’s encephalopathy (HE), are subjected to structured diagnostic work-ups guided by established criteria for autoimmune encephalitis (AE) (8), autoimmune psychosis (9), and the Antibody Prevalence in Epilepsy and Encephalopathy (APE2) score (10).

### Participants

Electronic medical records from January 2022 to January 2025 were systematically screened using ICD-10 diagnostic codes for schizophrenia-spectrum disorders (F20–F29) and major affective disorders, including depressive disorders (F30–F39). The initial cohort comprised 484 female patients (aged ≥18 years) hospitalized with these disorders who had undergone routine serological testing for thyroid autoantibodies, anti-thyroid peroxidase (anti-TPO, 0–34 IU/mL) and anti-thyroglobulin (anti-TG, 0–115 IU/mL) during their initial admission.

Inclusion criteria required a positive thyroid antibody result, defined as either anti-TPO (0–34 IU/mL) or anti-TG (0–115 IU/mL) serum titer exceeding the institutional laboratory’s reference range. Exclusion criteria included incomplete medical records, indeterminate final diagnoses, unrecorded antibody test results, or documented clinical hypo- or hyperthyroidism. After applying these criteria, the final study cohort consisted of 40 female psychiatric inpatients who were antibody-positive.

### Diagnostic assessments and procedures

Psychiatric diagnoses and neuropsychiatric syndrome features were confirmed by senior psychiatrists, using DSM-5 criteria. Standardized rating scales were prospectively documented in each patient’s chart during hospitalization to assess symptom severity, cognitive status, and functional impairment, including:

- Neuropsychiatric Inventory (NPI): To evaluate the severity and range of neuropsychiatric symptoms (e.g., delusions, hallucinations, agitation, mood lability) (11).
- Bush–Francis Catatonia Rating Scale (BFCRS): To screen for and quantify catatonic signs (12).
- Confusion Assessment Method (CAM): To identify delirium (acute confusional state) in patients with fluctuating consciousness or inattention (13).
- Montreal Cognitive Assessment (MoCA): To assess global cognitive function, administered at baseline and follow-up for patients with suspected cognitive impairment or encephalopathy (14).
- Global Assessment of Functioning (GAF): To rate overall psychosocial and occupational functioning on a 0–100 scale (lower scores indicate worse functioning) (15).
- Clinical Global Impression–Severity (CGI-S) and Improvement (CGI-I): To gauge illness severity at admission and improvement by discharge, respectively (16).

For patients with confirmed HE, MoCA assessments were conducted before and after immunotherapy to objectively document changes in cognition and behavior associated with the treatment.

### Neurodiagnostic work-up

Patients suspected of having an underlying autoimmune encephalopathy underwent an extensive neurodiagnostic evaluation as part of the standard inpatient protocol, including:

- Lumbar Puncture and CSF Analysis: Cerebrospinal fluid (CSF) was analyzed for signs of inflammation or infection (e.g., lymphocytic pleocytosis, elevated protein, IgG index, oligoclonal bands) and tested for neuronal and onconeuronal autoantibodies (e.g., anti-NMDAR, LGI1, GABABR) to exclude known antibody-mediated encephalitides.
- Electroencephalography (EEG): Routine EEG was performed to detect diffuse slowing or epileptiform activity indicative of encephalopathy.
- Neuroimaging: Structural brain imaging with magnetic resonance imaging (MRI) was conducted for all patients, with findings expected to be normal or show nonspecific changes in HE cases.
- Functional Imaging: In selected cases with diagnostic uncertainty, 18F-fluorodeoxyglucose positron emission tomography (FDG-PET) was used to assess cerebral glucose metabolism, identifying patterns of cortical hypometabolism consistent with autoimmune encephalopathy.

### Autoimmune and extended medical workup

In cases with suspected autoimmune involvement, both central nervous system–specific and systemic autoimmune markers were evaluated. CSF and serum samples were tested for a panel of neuronal autoantibodies associated with AE, including NMDAR, LGI1, CASPR2, GABABR, AMPAR, DPPX, and antibodies against onconeural antigens, amphiphysin, CV2.1, Ma2/Ta, Ri/ANNA-2, Yo/PCA-1, Hu/ANNA-1, Recoverin, SOX1 (AGNA), Zic4, GAD 65. Onconeural antibodies were detected by the combined use of Line immunoassay, and when positive, it is confirmed using indirect immunofluorescence. Cell surface antibodies were investigated only by commercial assays using fixed cells.

Systemic autoimmune screening was conducted for antinuclear antibodies (ANA), extractable nuclear antigen (ENA) panel, anti-dsDNA, ANCA, antiphospholipid antibodies, rheumatoid factor (RF), and complement levels (C3, C4) to exclude conditions such as systemic lupus erythematosus, Sjogren’s syndrome, antiphospholipid antibody syndrome and ANCA vasculitis.

CSF analysis also involved microbiological testing (e.g., Gram staining, culture, PCR for HSV-1/2, VZV, CMV, EBV, enteroviruses, HHV-6) to rule out infectious causes of encephalitis. Thoracoabdominopelvic contrast-enhanced computed tomography (CT) screened for occult malignancies, and cranial CT angiography was performed in cases where vasculitic processes were suspected.

All patients index episode was characterized with subacute onset neuropsychiatric features. Extended work-up was conducted during the patients’ index episode. Illness duration reflects the interval from the patient’s initial psychiatric diagnosis to the onset of neuropsychiatric manifestations that prompted referral to our center for extended diagnostic evaluation.

### Diagnostic criteria and case classification

Final neuropsychiatric diagnoses were determined by a multidisciplinary team of psychiatrists and neurologists and documented in discharge summaries. Patients were classified into HE or non-HE groups based on the diagnostic criteria proposed by Graus et al. (2016) (8) and revised Graus criteria (9) for HE.

**Figure 1:**
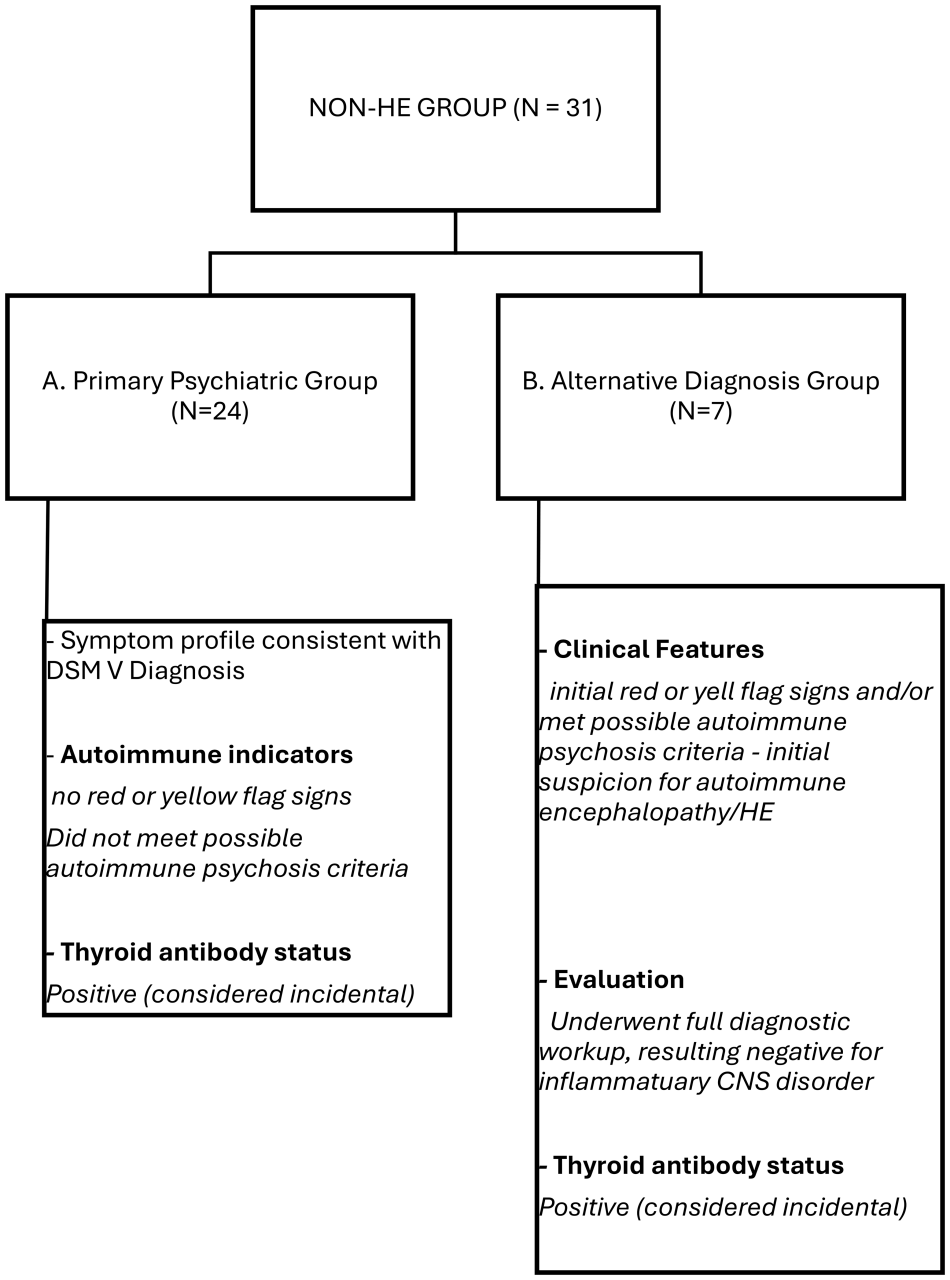
Diagnostic assessment of anti-thyroid antibody positive patients.

All patients in HE group fully met the thyroid-function requirement of the Graus diagnostic criteria for HE. For classification purposes, individuals who initially presented with a clinical suspicion of an autoimmune condition (such as Hashimoto’s Encephalopathy) but were ultimately assigned a different, non-autoimmune diagnosis based on comprehensive clinical, laboratory, and imaging assessments, were categorized into the Alternative Diagnosis (AD) group (**Figure 1**). We adopted a two-step algorithm. Step 1 involved universal antibody testing on admission (yield: 8.3% seropositive). Step 2 triggered a neurological evaluation—CSF analysis, EEG, MRI, ^18F-FDG-PET, and neuropsychological testing—for patients with “yellow or red flag” criteria (cognitive decline, fluctuating consciousness, catatonia) or Pollak et al.’s autoimmune psychosis framework. Of 40 seropositive patients, sixteen advanced to Step 2, nine confirmed as HE, yielding 56% in the high-suspicion subgroup.

A subgroup analysis was conducted for patients initially suspected of HE or autoimmunity but ultimately diagnosed with alternative conditions, to explore potential overlaps or distinguishing features. Among the patients classified as non-Hashimoto encephalopathy (non-HE), patients classified into AD subgroup had elevated anti-thyroid peroxidase or anti-thyroglobulin titers and displayed at least one “yellow/red-flag” indicator—or fulfilling the proposed *possible autoimmune psychosis* criteria—suggesting an autoimmune neuropsychiatric process, and (iii) were euthyroid or had only subclinical thyroid-function abnormalities, thereby phenotypically overlapping with the strict HE group. In contrast, the other non-HE patients exhibited incidental anti-TPO/anti-TG positivity; their neuropsychiatric presentations were fully attributable to established primary psychiatric disorders, and none displayed clinical “yellow/red-flag” features suggestive of autoimmune involvement, and therefore they did not undergo the extended diagnostic work-up. Each patient assigned to the Alternative Diagnosis (AD) subgroup underwent an extended diagnostic work-up—comprising detailed clinical history, comprehensive serological and infectious panels, brain MRI, EEG, and cerebrospinal-fluid analysis—to rule out possible autoimmune condition. The final comprehensive assessment identified an alternative, non–Hashimoto encephalopathy etiology that the prevailing neuropsychiatric manifestations were more likely attributable to an alternative diagnosis. Patients with systemic autoimmune disorders that could account for the neuropsychiatric presentation (e.g., systemic lupus erythematosus, multiple sclerosis, Behçet disease, sarcoidosis, antiphospholipid antibody syndrome, Sjogren’s syndrome) were excluded, as were individuals receiving any immune-modulating therapy. Patients also had undergone an extended medical work-up (comprehensive history, serological autoantibody panel, and targeted neuroimaging) in order to verify that none of these conditions were present in the final Hashimoto encephalopathy cohort.

### Treatment and outcomes

Clinical response to corticosteroid treatment was assessed retrospectively via daily progress notes and discharge summaries. Response was defined as neuropsychiatric improvement temporally associated with steroid initiation, evidenced by resolution of delirium (per CAM), reduction in catatonic or psychotic symptoms, cognitive improvement (per MoCA), and functional recovery (per GAF/CGI-I). In HE cases, response typically emerged within days to weeks of initiating high-dose intravenous methylprednisolone (500–1000 mg/day for 5–7 days). 31 patients constituting the non-HE group did not receive empirical steroids or any other form of immunotherapy. Their treatment adhered to current, evidence-based practice guidelines aligned with each patient’s final diagnosis. No empirical ‘steroid challenge’ was administered outside the HE group, therefore no patient was reassigned to an alternative-diagnosis group on the basis of steroid non-responsiveness.

### Data extraction and statistical analysis

Demographic, clinical, paraclinical, treatment, and outcome variables were extracted from structured electronic records. Continuous data are reported as mean ± SD or median (IQR) depending on normality (Shapiro–Wilk test). Categorical data are expressed as counts and percentages. Group comparisons employed Chi-square or Fisher’s exact tests for categorical variables and independent-samples t-tests or Mann–Whitney U tests for continuous variables, as appropriate. Because the HE group was small (n = 9), analyses were descriptive; multivariable modelling and diagnostic sensitivity/specificity calculations were not performed. Two-tailed p < 0.05 denoted statistical significance. Analyses were performed in IBM SPSS Statistics v27.

### Ethical considerations

The Ankara University Institutional Review Board approved the study (No. 2025/217), and waived written consent owing to its retrospective design. All data were de-identified and analyzed in compliance with the Declaration of Helsinki and STROBE reporting guidelines.

## Results

### Sample characteristics and diagnostic classification

Between January 2022 and January 2025, 484 female psychiatric in-patients were screened. Forty individuals (8.3%) tested positive for anti-thyroid antibodies and satisfied the study’s inclusion criteria. Of these, nine (22.5%) fulfilled diagnostic criteria for Hashimoto’s encephalopathy (HE), whereas 31 (77.5%) were classified as non-HE. Within the non-HE cohort, 28 patients carried primary psychiatric diagnoses, and three received alternative neurological or autoimmune diagnoses (e.g. N=1/31 paraneoplastic autoimmune encephalitis or N=2/31 neurodegenerative disease). All patients in HE group had anti-TPO levels above 200 IU/mL.

At admission, the HE and non-HE groups did not differ significantly in mean age (49.9 ± 11.1 years *vs*. 46.0 ± 12.6 years; *p*=0.446). In contrast, HE patients showed a significantly later onset of psychiatric symptoms (42.9 ± 13.0 years *vs*. 30.1 ± 11.2 years; *p*=0.011) and a shorter interval between initial psychiatric diagnosis and index presentation (7.0 ± 9.3 years *vs*. 16.0 ± 11.2 years; *p*=0.009). Functional impairment was greater among HE patients, reflected by lower Global Assessment of Functioning scores (31.7 ± 5.0 *vs*. 51.6 ± 12.6; *p* < 0.001), higher Neuropsychiatric Inventory totals (53.8 ± 13.2 *vs*. 22.9 ± 14.8; *p* < 0.001), and higher Clinical Global Impression–Severity ratings (6.7 ± 0.5 *vs*. 4.2 ± 1.2; *p* < 0.001). Sociodemographic and clinical details are presented in **Table 1**.

**Table 1:** Sociodemographic and clinical features of patients with primary psychiatric disorders *vs*. Hashimoto’s encephalopathy.

Variable	Non-HE group (n = 31)	Hashimoto’s Encephalopathy (n = 9)	Test statistic	p-value
Age (years)	45.97 ± 12.58	49.89 ± 11.06	*U*=116.0 (Z=–0.762)	0.446
Age at onset (years)	30.10 ± 11.18	42.89 ± 13.04	*U*=59.0 (Z=–2.625)	0.011
Initial psychiatric diagnosis - index presentation interval (years)	16.00 ± 11.22	7.00 ± 9.35	*U*=61.0 (Z=–2.545)	0.009
GAF score (baseline)	51.61 ± 12.61	31.67 ± 5.00	*U*=29.0 (Z=–3.704)	< 0.001
NPI score (baseline)	22.94 ± 14.79	53.78 ± 13.17	*U*=19.0 (Z=–3.965)	< 0.001
CGI-S (baseline severity)	4.19 ± 1.22	6.67 ± 0.50	*U*=15.0 (Z=–4.119)	< 0.001
CGI-I (post-tx improvement)	1.94 ± 0.96	1.44 ± 0.53	*U*=102.0 (Z=–1.311)	0.190

Data are presented as mean ± standard deviation. *U*=Mann–Whitney U test (two-tailed). GAF, Global Assessment of Functioning; NPI, Neuropsychiatric Inventory; CGI-S, Clinical Global Impression–Severity; CGI-I, Clinical Global Impression–Improvement. p < 0.05 indicates statistical significance.

Patients with systemic or organ-specific autoimmune diseases capable of producing primary neuropsychiatric or central nervous system involvement—such as systemic lupus erythematosus, multiple sclerosis, Behçet disease, sarcoidosis, autoimmune vasculitides, or antiphospholipid syndrome—were explicitly excluded, as were individuals receiving ongoing immunomodulatory therapies. Consequently, none of the 40 enrolled patients (neither the nine in the HE group nor the 31 in the non-HE group) had a co-existing autoimmune condition presenting with neuropsychiatric signs and symptoms. No patient in either group harbored a co-existing active autoimmune disorder that required ongoing active treatment. Within the HE cohort (n = 9), co-existing autoimmune disorders comprised thyroiditis by history and thyroid ultrasonography or scintigraphy (n = 7). Coeliac disease (n = 1), rheumatoid arthritis (n = 1), and tracheobronchial asthma (n = 1) were identified by history. In the non-HE cohort (n = 31), autoimmune thyroiditis (n = 3), rheumatoid arthritis (n = 1), and asthma (n = 1) were documented by history. None of these comorbidities required active immunosuppressive treatment during the study period.

According to the Graus 2016 proposed criteria, five patients in the HE group also fulfilled the criteria for probable NMDAR encephalitis (Cases 1, 2, 3, 4, and 7). Four patients met the criteria of possible autoimmune encephalitis (Cases 2, 7, 8, and 9); of these, two (Cases 2 and 7) fulfilled criteria for both the possible AE and probable NMDAR encephalitis. Notably, one patient (Case 7) met the criteria for bot probable but antibody-negative **AE** and probable NMDAR encephalitis.

### Neuropsychiatric syndrome patterns and clinical characteristics of HE patients

Complex and atypical neuropsychiatric presentations characterized the HE cohort. Delirium occurred in 88.9% of HE patients versus 6.5% of non-HE group (χ² = 25.281, *p* < 0.001). Catatonia was documented in 77.8% of HE cases (7/9) compared with 32.3% of non-HE cases (10/31) (χ² = 5.914, *p*=0.015). Rates of psychosis (88.9% *vs*. 61.3%), mania (44.4% *vs*. 45.2%), and depressive syndromes (22.2% *vs*. 38.7%) did not differ significantly (χ² ≤ 0.833, *p* ≥ 0.361 for all).

On formal cognitive testing, nearly all HE patients displayed marked impairment; Montreal Cognitive Assessment (MoCA) scores frequently fell below 10/30, and most of the patients (N=8/9) patients were unable to complete basic items during the acute phase. Delusions were likewise prevalent (88.9%), often taking paranoid or bizarre forms (e.g., Capgras- or Cotard-like). Hallucinations occurred in all patients in HE group (N=9). In eight of cases hallucinations were a manifestation of an established delirum syndrome at presentation.

### Neurodiagnostic findings

Evidence of neuroinflammation in cerebrospinal fluid—lymphocytic pleocytosis, elevated IgG index, or type-2 oligoclonal bands—was detected in 55.6% of HE patients (N=5/9) but in none of the non-HE patients (0/31). All HE cases were negative for established neuronal autoantibodies; one non-HE patient harbored a paraneoplastic antibody, leading to a diagnosis of paraneoplastic encephalitis rather than HE.

**Figure 2:**
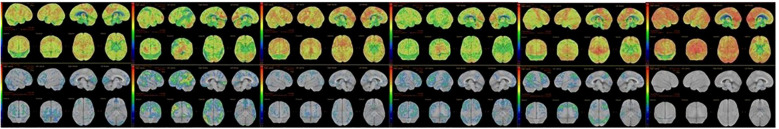
FDG-PET hypometabolism in six HE patients.

Diffuse EEG slowing was documented in 33.3% of HE patients (*p*=0.001). Structural MRI findings were nonspecific for limbic encephalitis and/or autoimmune encephalitis in the context of bilateral medial temporal hyperintensities in both cohorts. Fluorodeoxyglucose positron emission tomography (FDG-PET) revealed cortical hypometabolism in 85.7% of imaged HE patients (6/7), who underwent FDG-PET imaging, compared with 0% of imaged AD patients (0/5) (χ² = 10.500, *p*=0.001). FDG-PET images of six HE patients are shown in **Figure 2**. Additionally, two AD patients instead showed patterns suggestive of neurodegeneration, and both were subsequently diagnosed with neurodegenerative disease (N=2/7). Neuropsychiatric syndrome patterns and neurodiagnostic features in patients with the non-HE group and the HE group are shown in **Table 2**.

**Table 2:** Neuropsychiatric syndrome patterns and neurodiagnostic features in non-HE *vs*. Hashimoto’s encephalopathy groups.

Neuropsychiatric syndrome patterns – DSM V	Non-HE (n=31)	HE (n=9)	χ²	p-value
Catatonic syndrome	10/31 (32.3%)	7/9 (77.8%)	5.914	0.015
Psychotic syndrome	19/31 (61.3%)	8/9 (88.9%)	2.422	0.120
Manic syndrome	14/31 (45.2%)	4/9 (44.4%)	0.001	0.970
Depression (syndromal)	12/31 (38.7%)	2/9 (22.2%)	0.833	0.361
Delirium	2/31 (6.5%)	8/9 (88.9%)	25.281	< 0.001
**Abnormal CSF**	0/7 (0%)	5/9 (55.6%)	5.657	0.017
**Abnormal EEG**	0/31 (0%)	3/9 (33.3%)	11.171	0.001
**Abnormal FDG-PET**	0/5 (0%)	6/7 (85.7%)	10.500	0.001
**Treatment resistance**†	0/31 (0%)	9/9 (100%)	40.000	< 0.001

Data are presented as a number of patients with a feature/number assessed in the group (%). χ², Chi-square test (two-tailed). CSF and FDG-PET were performed in subsets of patients with diagnostic uncertainty (n=16 for CSF analysis, n=14 for FDG-PET). LP, lumbar puncture; EEG, electroencephalography; FDG-PET, fluorodeoxyglucose positron emission tomography.

†Treatment resistance is defined as a lack of clinically significant improvement with standard psychiatric treatment, necessitating immunotherapy. Abnormal CSF: nonspesific but indicating inflammation. Abnormal EEG: nonspesific abnormal slowing. Abnormal FDG-PET: nonspesific but atypical hypometabolism.

### Subgroup of suspected autoimmune cases

Sixteen anti-thyroid antibody positive patients were initially evaluated for possible HE or autoimmune encephalopathy (N=16/40). After full work-up, nine were confirmed as HE and seven were classified into alternative diagnosis (AD; neurodegenerative disorders, N=2; definite autoimmune encephalitis, N=1; primary psychiatric disorder, N=3). Delirium distinguished confirmed HE from false-positive cases (88.9% *vs*. 14.3%; *p*=0.003), whereas catatonia was frequent in both subgroups (77.8% *vs*. 100%; *p*=0.182). Psychosis, mood syndromes, and abnormal EEG did not differ significantly (p < 0.05). All HE patients were resistant to standard psychiatric therapies involving ECT and/or lorazepam trials for their catatonic symptoms and antipsychotics and/or mood stabilator for their psychotic and manic syndromes, and required corticosteroids; all AD patients improved with conventional treatment for their catatonic symptoms, even patients diagnosed with neurodegenerative disease improved significantly with ECT treatment, except one patient with paraneoplastic autoimmune encephalitis. and paraneoplastic encephalitis responded well (*p* < 0.001). Subgroup comparison regarding neurodiagnostic syndrome patterns and paraclinical features is shown in **Table 3**.

**Table 3:** Subgroup comparison – patients confirmed with Hashimoto’s encephalopathy *vs*. those initially suspected of HE but classified into an alternative diagnosis.

Clinical and Paraclinical features	AD (n=7)	HE (n=9)	Test statistic	p-value
Age (years)	46.43 ± 11.87	46.89 ± 13.04	*U*=31.0	0.958
Age at onset (years)	33.71 ± 10.89	42.89 ± 13.04	*U*=24.5	0.458
Illness duration (years)	12.71 ± 9.87	7.00 ± 9.35	*U*=16.0	0.097
GAF score	48.57 ± 13.21	31.67 ± 5.00	*U*=21.0	0.142
NPI score	25.71 ± 15.34	53.78 ± 13.17	*U*=9.0	0.014
CGI-S	4.43 ± 1.13	6.67 ± 0.50	*U*=13.5	0.039
CGI-I	2.14 ± 1.07	1.44 ± 0.53	*U*=14.5	0.047
**Clinical and paraclinical features**	**AD (n=7)**	**HE (n =9)**	**χ²**	**p-value**
Catatonic syndrome	7/7 (100%)	7/9 (77.8%)	1.778	0.182
Psychotic syndrome	6/7 (85.7%)	8/9 (88.9%)	0.036	0.849
Manic syndrome	5/7 (71.4%)	4/9 (44.4%)	1.165	0.280
Depression	1/7 (14.3%)	2/9 (22.2%)	0.163	0.687
Delirium	1/7 (14.3%)	8/9 (88.9%)	8.905	0.003
Abnormal CSF*	0/7 (0%)	5/9 (55.6%)	5.657	0.017
Abnormal EEG	0/7 (0%)	3/9 (33.3%)	2.872	0.090
Abnormal FDG-PET*	0/5 (0%)	6/7 (85.7%)	10.500	0.001
Treatment resistance†	0/7 (0%)	9/9 (100%)	16.000	< 0.001

Suspected HE, patients initially suspected of HE but ultimately diagnosed with a primary psychiatric disorder; Confirmed HE, patients with a confirmed Hashimoto’s Encephalopathy diagnosis. Data are mean ± SD or n/N (%) as indicated. *U*=Mann–Whitney U test; χ², Chi-square test (two-tailed). Abnormal CSF and FDG-PET were assessed only in those who underwent the procedures (subset of the 14 suspected HE patients). Abbreviations as in previous tables. **p** < 0.05 indicates statistical significance. Abnormal CSF, nonspesific but indicating inflammation; Abnormal FDG-PET: nonspesific but indicating atypical hypometabolism.

Neurological signs unique to the HE group included stroke-like focal deficits (11.1%), orofacial dyskinesias (11.1%), dysarthria (44.4%), parkinsonian tremor/rigidity (22.2%), myoclonus (22.2%), stroke-like features-unilateral limb paralysis (11.1%), and language disorder (55.5%) A comprehensive list of neurologic neuropsychiatric signs in HE patients are presented in **Table 4**.

**Table 4:** Neuropsychiatric and neurologic signs and symptoms in confirmed Hashimoto’s encephalopathy patients (n=9).

Clinical signs and symptoms	Number of patients (n=9)	Percentage (%)
Cognitive impairment	9	100%
Catatonic signs and symptoms	7	77.8%
Delusions	8	88.9%
Hallucinations	9	100%
Depressive symptoms	2	22.2%
Delirious features	8	88.9%
Neurological signs:		
- Language disorder - Myoclonus	5 2	55.5% 22.2%
– Parkinsonism	2	22.2%
– Orofacial dyskinesia	2	22.2%
– Dysarthria	4	44.4%
– Seizures	0	0%
– Stroke-like episodes	1	11.1%

CSF, cerebrospinal fluid; EEG, electroencephalography; FDG-PET, fluorodeoxyglucose positron emission tomography.

### Treatment response and outcomes

All the patients in the HE group failed to improve with antipsychotics, mood stabilizers, lorazepam, or electroconvulsive therapy (ECT), necessitating high-dose intravenous methylprednisolone (1 g day¹ for 5–7 days). Corticosteroid therapy produced rapid resolution of catatonia, delirium, psychosis, mood disturbance, and cognitive impairment, typically within 3–7 days. The BFCRS score of all patients in the HE group was reduced to 0.

**Figure 3:**
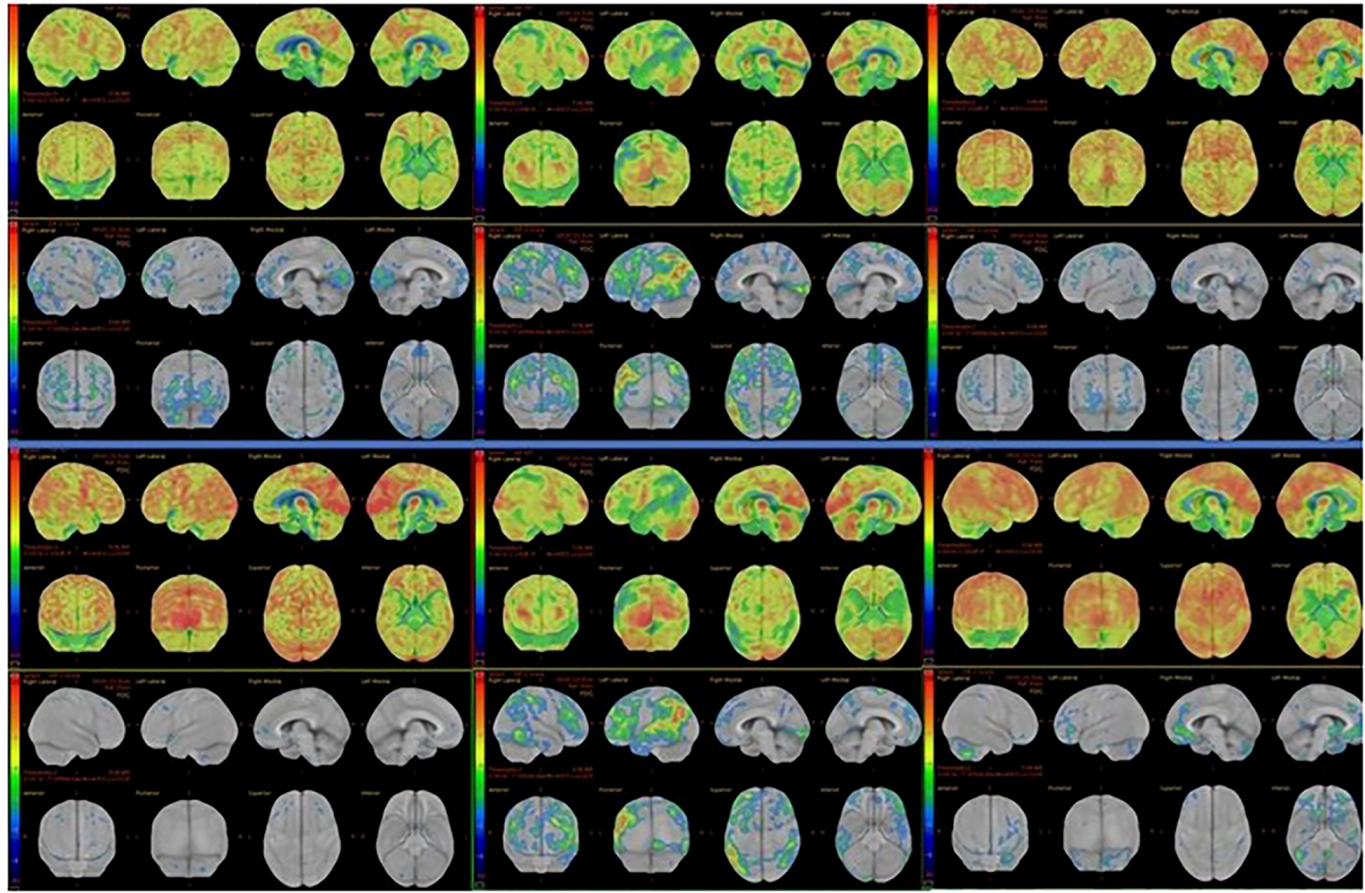
Treatment response in three patients with HE.

In the non-HE group initially suspected of HE (AD group), lorazepam and ECT effectively treated catatonia and other psychiatric symptoms; even those later diagnosed with neurodegenerative disease responded to ECT for their catatonic signs and symptoms. By discharge, Clinical Global Impression–Improvement scores did not differ between HE and primary psychiatric patients (1.44 ± 0.53 *vs*. 1.94 ± 0.96; *U*=102.0, *p*=0.190), reflecting substantial symptomatic recovery in both cohorts. In the HE group, MoCA scores improved from severely impaired (<10/30) to near-normal ranges (mid-20s to 30/30), and associated neurological signs, including parkinsonism, myoclonus, stroke-like features, dyskinesia, dysarthria, and language disorder, resolved in parallel. The FDG‐PET scans obtained during the remission phase following initial therapy for three patients are presented in **Figure 3**. Early control FDG‐PET studies performed after acute immunotherapy showed improvements in the pretreatment hypometabolism for these three patients. No follow-up FDG-PET data are available for other patients, as control scans were not performed. In those with baseline EEG abnormalities (N=3), follow‐up EEGs normalized. Notably, the subacute neurological motor symptoms listed in **Table 4**—Parkinsonism, myoclonus, dysarthria, language disorder, and stroke‐like episodes—resolved after 5–7 days of pulse therapy, concomitant with improvement in other neuropsychiatric manifestations. In the management of HE, following initial pulse corticosteroid therapy, immunotherapy was arranged as follows: two patients received maintenance treatment with oral steroids and azathioprine for nine months, one patient received maintenance oral steroids for six months, and the remaining patients received maintenance oral steroids for two months. Detailed objective treatment outcomes of patients with HE are shown in **Table 5**.

**Table 5:** Objective treatment outcomes in the HE group.

Case	MoCA score (Pre → Post)	FDG-PET(Post-Treatment)	EEG follow-up	Maintenance therapy	Relapse	Response to relapse	Final outcome
1	<10 → 28	improvement		Steroids + AZA (9 mo)	Yes (1 mo. after AZA stopped)	Resistance to Re-pulse steroids, Marked improvement with IVIG	Asymptomatic at 3 yrs
2	<10 → 21	improvement	Normalized	Steroids + AZA (9 mo)	Yes (2 mo after steroids stopped)	Improvement with Re-pulse steroids	Asymptomatic at 3 yrs
3	<10 → 24	improvement	Normalized	Steroids (6 mo)	Yes (1 year after steroid cessation)	Improvement with Re-pulse steroids	Asymptomatic at 1 year
4	<10 → 27	Not performed		Steroids (2 mo)	No	—	Sustained remission
5	<10 → 29	Not performed		Steroids (2 mo)	No	—	Sustained remission
6	<10 → 30	Not performed		Steroids (2 mo)	No	—	Sustained remission
7	<10 → 25	Not performed		Steroids (2 mo)	No	–	Sustained remission
8	<10 → 26	Not performed		Steroids (2 mo)	No	—	Sustained remission
9	<10 → 27	Not performed	Normalized	Steroids (2 mo)	No	—	Sustained remission

CSF, cerebrospinal fluid; EEG, electroencephalography; FDG-PET, fluorodeoxyglucose positron emission tomography.

During post‐treatment follow‐up, three patients experienced relapse within the first year of their follow-up. Among the three patients who relapsed, the timing of relapse was as follows: one patient relapsed within one month of discontinuing azathioprine; a second patient relapsed within two months after steroid therapy cessation; and the third patient relapsed at one year following two months of discontinuation of oral steroids. Among these patients, two achieved complete remission following pulse corticosteroid therapy. Thereafter, long-term maintenance immunosuppression with oral corticosteroids and azathioprine was initiated, and both individuals have remained asymptomatic at three-year follow-up. In contrast, the third patient’s relapse proved refractory to corticosteroid therapy; after remission induction with intravenous immunoglobulin (IVIG), immunomodulatory treatment was initiated.

In sum, among thyroid-antibody–positive psychiatric in-patients (N=40/484), a distinct subset (22.5%) met criteria for Hashimoto’s encephalopathy. These patients exhibited late-onset, acute delirium and/or catatonia, profound functional impairment, objective markers of neuroinflammation, and resistance to standard psychiatric care. Prompt recognition of red-flag features—especially unexplained delirium, concurrent catatonia, and treatment resistance—combined with targeted investigations (thyroid antibodies, CSF analysis, EEG, FDG-PET) enabled timely diagnosis and corticosteroid treatment, resulting in excellent clinical outcomes. Detailed descriptions of FDG-PET, CSF, EEG and MRI investigations in HE patients are shown in **Table 6**.

**Table 6:** Neurodiagnostic findings in HE group.

Case	FDG-PET	CSF	EEG	MRI
1	Severe Diffuse hypometabolism in bilateral cerebral hemispheres, prominent in parietal, temporal, and occipital lobes.	Cell= 5 WBC /mm ³, Oligoclonal bands (OCB) Type 2	Normal trace, 8–9 Hz Background rhythm	Normal
2	Severe Diffuse hypometabolism in bilateral frontal, parietal, temporal, and occipital lobes, most prominently in the left inferior parietal and temporal lobes, with low-level hypometabolism in the posterior cingulate gyrus.	Cell= 0 WBC/mm^3;^ OCBs (Type IV)	6–7 Hz background rhythm, Abnormal diffuse slowing	Nonspecific
3	Severe Diffuse hypometabolism in bilateral frontal, parietal, temporal, and occipital lobes (prominently in the right frontal region), anterior cingulate gyrus, and primary visual cortex.	Cell=0 WBC/mm ^3^; OCBs (Type IV)	5–6 Hz Background rhythm,Abnormal diffuse slowing.	Normal
4	N/A.	Cell=0 WBC/mm^3^;Protein=53.3 mg/dL; IgG=67.3 mg/dL,; IgG=index 0.89; OCBs (Type II and Type III)	Normal trace, 9–10 Hz background rhythm	T2-FLAIR periventricular hyperintensities compatible with inflammatory lesions.
5	Normal metabolism	Cell=5 WBC/mm^3^; IgG index=1.02	Normal Trace, 8–9 Hz Background rhythm	Normal
6	Minimal relative focal hypometabolism in the left temporal and lateral prefrontal cortex, and left inferior parietal lobe compared to the contralateral hemisphere.	Cell= 0 WBC/mm^3^; OCBs type IV	Normal trace, 9–10 Hz Background rhythm	Normal
7	N/A.	Cell: 10 WBC/mm³.	Normal trace, 9–10 Hz Background rhythm	Normal
8	Severe patchy/regional hypometabolism in multiple areas of bilateral cerebral hemispheres, including the inferior parietal lobe, occipital lobe, lateral temporal lobe, precuneus, and lateral prefrontal cortex (Diffuse hypometabolic focal areas in bilateral cerebral cortex)	Cell=1 WBC mm^3^ IgG index=0.91	Normal Trace, 8–9 Hz background rhythm	Normal.
9	Severe diffuse hypometabolic focal areas in bilateral cerebral hemispheres.	Cell=0 WBC/mm^3^	5–6 Hz background rhythm, abnormal diffuse slowing	Normal.

CSF, cerebrospinal fluid; EEG, electroencephalography; FDG-PET, fluorodeoxyglucose positron emission tomography.

## Discussion

This study is, to our knowledge, the first to systematically and structurally evaluate Hashimoto’s encephalopathy (HE) in psychiatric inpatients through a retrospective chart review of cases that had been prospectively assessed at the time of admission. Conducted in a specialized women’s neuropsychiatry unit, all included patients underwent a structured, protocol-driven diagnostic work-up during their index hospitalization, incorporating multimodal paraclinical assessments—CSF analysis, EEG, MRI, and FDG-PET—together with standardized clinical and cognitive tools such as MoCA, BFCRS, and CAM. This comprehensive, protocolized approach addresses a major gap in the literature, as previous HE studies have predominantly focused on neurology cohorts or relied on retrospective data without structured, prospective psychiatric evaluation. By integrating real-time standardized assessments with retrospective outcome analysis, our study underscores the importance of thorough neuropsychiatric evaluation for accurate HE diagnosis in patients whose presentations are dominated by psychiatric symptoms. Although HE was reported relatively uncommon — In the present study, HE accounted for 1.9% of all admissions and 22.5% of anti-thyroid antibody positive cases, — it emerged as a distinct, severe, and steroid-responsive neuropsychiatric syndrome. The systematic multimodal approach enabled identification of key clinical, cognitive, and neurodiagnostic features that differentiate HE from primary psychiatric disorders, facilitating earlier diagnosis and targeted intervention in this otherwise elusive condition.

### Diagnostic challenges in HE

Anti-thyroid (TPO, TG) antibodies are common (8–13%) in the general population and even more prevalent in psychiatric patients. In our cohort of 40 anti-thyroid antibody positive inpatients, only 9 (22.5%) met strict HE criteria – far above the 5.3% and 15.2% reported in neurology cohorts (17, 18). This likely reflects our focus on atypical, treatment-resistant cases. As Valencia-Sanchez et al. (2021) (19) observed, most seropositive patients did not have HE, underscoring that thyroid antibodies alone have limited specificity and that HE may be under-recognized in psychiatric settings.

In the present study, chart reviews revealed prior psychiatric symptoms treated elsewhere, but we did not consider these as causal evidence for Hashimoto’s encephalopathy (HE). HE has been reported to be characterized with a chronic or relapsing–remitting course, which may explain the long interval between patients’ earliest psychiatric presentation and referral to our center, without implying causality. All patients received a comprehensive autoimmune and neuropsychiatric evaluation during the index episode, characterized by subacute onset of neuropsychiatric symptoms.

Given HE’s often chronic or relapsing-remitting course—unlike the typically monophasic trajectory of other antibody-mediated encephalitides, such as NMDAR encephalitis—”mean disease duration” reflected the interval since the first documented psychiatric diagnosis in present study. In the HE group, the reported disease duration of our genuine HE patients represents the interval from the first documented psychiatric diagnosis to the index presentation. While all index episodes of the patients began with subacute neuropsychiatric symptoms, patients’ prior psychiatric history may reflect either early, unrecognized manifestations of HE, or alternatively, co-occurring functional psychiatric syndromes. Notably, in some cases, early signs of autoimmune dysfunction may have been misattributed to functional psychiatric illnesses, such as bipolar disorder, thereby delaying appropriate recognition and intervention.

We adopted a two-step diagnostic algorithm, which optimized sensitivity while reducing unnecessary invasive procedures among patients ultimately diagnosed with primary psychiatric disorders. Nevertheless, this strategy revealed spectrum bias, as the detection rate could have decreased substantially had the remaining 24 anti-thyroid antibody positive patients undergone equivalent evaluations. Importantly, stratifying cases into an initially suspected HE group (e.g., HE-like AD subgroup) underscores that anti-thyroid antibody positivity alone—even when accompanied by overlapping HE-like features—may be insufficient to establish a definitive HE diagnosis or to justify empirical immunotherapy in the absence of additional supportive evidence.

Absolute TPO/TG titers were omitted as they do not predict diagnosis or steroid responsiveness (5, 19). Antibody status was treated categorically to avoid causal inferences from incidental serology. Our findings echo previous warnings (4, 19) that thyroid antibodies must be interpreted within a multimodal framework of (i) compatible clinical features, (ii) neurodiagnostic abnormalities, and (iii) therapeutic response.

### Clinical phenotype: a catatonia–delirium complex

All nine HE patients had acute or sub-acute encephalopathy with severe cognitive and behavioral disturbances. Catatonia occurred in 77.8% and delirium in 88.9%, frequencies rarely seen in neurologic cohorts. Classical series note seizures, myoclonus, or ataxia (3, 7, 18), but our patients, from a psychiatric ward, exhibited catatonia, psychosis, or mood disturbances without epileptic features. Although catatonia has historically been underrecognized in Hashimoto’s Encephalopathy (HE), several case reports documented steroid-responsive catatonia as a manifestation of an autoimmune encephalopathy similarly noted catatonia in patients with HE (4, 20–25).

Catatonia (77.8%) and delirium (88.9%) co-occurred prominently in HE patients, marking a novel observation in this cohort and almost absent in the comparison group. While psychosis and mania were common in both groups, these findings suggest that the catatonia–delirium complex may indicate autoimmune encephalopathy/probable HE in psychiatric settings. Although psychosis is often reported (> 50%) in HE (3, 5), our data suggest that catatonia–delirium syndrome may be more informative diagnostically in certain populations. These findings highlight the value of systematic screening: in our unit, routine use of the Bush–Francis Catatonia Rating Scale (BFCRS) and the Confusion Assessment Method (CAM) enabled us to detect catatonia–delirium syndromes that might otherwise be missed.

Psychosis, while common, isn’t a reliable discriminator since it appears in both groups. Its prominence may mask catatonia and cognitive impairment, particularly in psychiatric units where catatonia is often under-assessed. This has important implications, as delayed recognition of HE due to psychosis can lead to postponed treatment. Additionally, severe cognitive impairment, commonly seen as global cognitive impairment, may indicate catatonia or delirium rather than primary decline. This overlap is rarely discussed in the literature, and standardized catatonia evaluations are seldom part of HE case series.

Catatonia may remain underdiagnosed in HE due to misclassification in neurology as “altered mental status,” confusion, or withdrawal, as well as latency in response, apraxia, motor incoordination, language impairments, behavioral disorganization, comprehension difficulties, akinesia, and bradykinesia. Terms like “mutism,” “bradykinesia,” “lack of responsiveness,” or “psychomotor retardation”—common in neurology notes—may describe catatonic states without labeling them (26). In our psychiatric unit, routine screening contributed to high prevalence rates. Based on this, we systematically applied BFCRS and the Confusion Assessment Method (CAM) at admission and during hospitalization.

Importantly, catatonia in HE was refractory to benzodiazepines and ECT but reversed with corticosteroids. By contrast, catatonia in anti-thyroid antibody positive patients with primary psychiatric or degenerative diagnoses responded to lorazepam or ECT alone. Clinically, failing to recognize catatonia–delirium is risky: misguided treatment (e.g., unnecessary antipsychotics) can precipitate malignant catatonia or neuroleptic malignant syndrome, and delirium may be misinterpreted as agitation. We therefore advocate routine BFCRS and CAM screening in anti-thyroid antibody positive, treatment-resistant patients. Catatonia alone was nonspecific: about one-third of anti-thyroid antibody positive cases showed catatonic signs responsive to benzodiazepines or ECT. The diagnostic weight lies in refractory catatonia–delirium alongside thyroid autoimmunity, negative neuronal antibody panels, and supportive paraclinical findings.

Our findings are among the first to quantify the prevalence of catatonia in a cohort of psychiatric inpatients who are positive for thyroid antibodies, suggesting that it may be a common and reversible phenotype when assessed systematically.

### Neurodiagnostic features

Neurodiagnostic testing is crucial for differentiating autoimmune encephalopathies, like probable HE (8), from primary psychiatric disorders, neurodegenerative, and neurodevelopmental disorders. Inflammatory CSF abnormalities (pleocytosis, elevated IgG index, oligoclonal bands) occurred in 55.6% of HE patients but in none of our anti-thyroid antibody positive psychiatric controls, consistent with autoimmune CNS inflammation. We observed no significant CSF protein elevation, aligning with recent reports that mild protein increases are nonspecific (3, 19). Many older studies reporting elevated CSF protein did not examine CSF neuronal antibodies (19). EEG showed diffuse slowing in 33.3% of HE patients (versus ~80% in older series), consistent with more recent cohorts (5, 27) (Chaudhuri et al., 2023; Mattozzi et al., 2020). Thus, EEG had modest sensitivity but, when abnormal, supported an encephalopathy diagnosis.

^18F-FDG-PET was the most sensitive modality in our sample, with cortical hypometabolism in 85.7% of HE cases. This aligns with evidence that FDG-PET can reveal functional abnormalities in autoimmune encephalopathy even when MRI and EEG are normal (19, 21, 28). In our study, the severity and extent of hypometabolism strongly suggested an autoimmune process. We therefore endorse including FDG-PET in the workup of anti-thyroid antibody positive, treatment-resistant psychiatric patients with unexplained cognitive decline.

Although only one patient in our HE group met the full criteria for antibody-negative probable autoimmune encephalitis (AE) as defined by Graus et al. (2016)—primarily due to normal MRI findings in the remaining patients—it remained important to consider that some patients may have exhibited a neuroinflammatory process independent of thyroid antibody status. Specifically, certain cases displayed laboratory features suggestive of central nervous system (CNS) inflammation, such as CSF abnormalities or EEG changes, This underscores the possibility that autoimmune etiology in some patients may be supported not solely by thyroid autoantibodies, but also by the presence of neuropsychiatric signs and symptoms associated with limbic dysfunction and other ancillary markers indicative of neuroinflammation. Hashimoto’s encephalopathy (HE), or steroid-responsive encephalopathy associated with thyroiditis (SREAT), is often linked to seropositive thyroid antibodies, which may suggest an autoimmune etiology but are not diagnostic on their own. they may support an autoimmune etiology as an epiphenomenon. Combined with atypical clinical presentations such as severe atypical cognitive dysfunction and other nonspecific but suggestive paraclinical evidence of CNS inflammation in cases with seropositive thyroid antibodies—such as severe cortical hypometabolism on FDG-PET, CNS-specific oligoclonal bands, elevated igG-index and EEG background slowing—thyroid antibodies can support the possibility of an immune-mediated process. Notably, although neither Case 5 nor Case 6 fulfilled any Graus criteria for autoimmune encephalitis, the atypical presentation with an elevated CSF IgG index in Case 5 and the delirium-independent severe cognitive impairment with FDG-PET hypometabolism in Case 6 constitute clinical–paraclinical cues that may indicate CNS autoimmunity and thus warrant initiation of empirical corticosteroid therapy.

Taken together, these observations emphasize that thyroid-antibody seropositivity should be interpreted in the broader context of converging clinical and paraclinical evidence for neuroinflammation. In our study empirical corticosteroids were initiated in individuals who also concurrently met proposed structured criteria for autoimmune encephalitides (e.g. possible AE, probable NMDAR encephalitis) and/or probable autoimmune psychosis – thereby strengthening the clinical rationale for immunosuppressive treatment. Inflammatory CSF markers (elevated IgG index, pleocytosis, or oligoclonal bands) emerged as important markers for the treatment initiation.

### Treatment response and prognosis

All nine HE patients responded rapidly to IV methylprednisolone (1 g/day for 5–7 days), with median CGI-I improving from “very much worse” at baseline to “very much improved” by day 7 and MoCA scores rising from <10 to 23–29. Parallel gains were seen on the BFCRS, CAM, and NPI, and follow-up EEG slowing and fronto-parietal ^18F-FDG hypometabolism normalized in follow-up testing in some patients.

Notably, two patients (22%) relapsed within four months of a rapid steroid taper; one regained remission after a second methylprednisolone pulse and extended prednisone taper, and the other after IVIG (2 g/kg) followed by azathioprine (2 mg/kg/day), with both remaining relapse-free at ≥ 12 months. These outcomes underscore the necessity of prolonged tapering and, where appropriate, long-term steroid-sparing therapy, paralleling the ~ 16% relapse rate in Laurent et al. (2016) and the taper-related relapses was also reported (27).

Our 100% steroid-response rate aligns with earlier series of HE but contrasts with varied outcomes in recent, broader cohorts. Castillo et al. (2006) (7) and Ferracci & Carnevale (2006) (29) reported nearly 100% remission with steroids, and other cohorts found 85–93% response rates (2, 30, 31). This treatment response correlates with earlier studies. Laurent et al. (2016) (3) reported a 91% improvement rate with corticosteroids in 251 HE cases. Figgie et al. (2024) (17) noted consistent steroid responsiveness in confirmed HE cases and recommended empirical steroid trials for unexplained neuropsychiatric presentations with thyroid antibodies. Figgie et al. (2024) observed rapid improvement in all confirmed HE patients (7/7) following the same steroid protocol. Dumrikarnlert et al. (2023) (18) found that 75% of patients exhibited significant improvement, while 16.6% showed no benefit, highlighting response variability. Chaudhuri et al. (2023) (28) noted initial steroid efficacy but warned of relapse risks, especially with rapid tapering or inadequate treatment.

In contrast, broader HE cohorts report much lower remission rates (5 19) (32%; 27% respectively). Mattozzi et al. (2020) discovered that only 31.6% of patients with classical HE criteria achieved full remission with steroids (5). Valencia-Sanchez et al. (2021) found that many initially suspected HE patients did not respond to steroids, with most non-responders receiving alternative diagnoses (19). Litmeier et al. (2016) (32) summarized the variability in response rates (32–93%) across the literature, suggesting possible HE subtypes. Thus, steroid responsiveness is supportive but not definitive for diagnosis. This discrepancy highlights how inclusion criteria affect perceived efficacy: our study, like the aforementioned high-response series, required (i) treatment resistance to standard psychotropics, (ii) objective neurodiagnostic abnormalities, and (iii) a monitored steroid trial to confirm HE.

Our results support corticosteroid responsiveness as a diagnostic adjunct in ambiguous or treatment-resistant cases, in line with Figgie et al.’s emphasis on the risks of indiscriminate steroid use (9). Initiating immunotherapy based solely on thyroid antibody positivity, without supportive paraclinical evidence (e.g., CSF inflammation or PET abnormalities), may result in misdiagnosis or iatrogenic harm, echoing warnings from Valencia-Sanchez et al. (2021) and Mattozzi et al. (2020) (5, 19). Their experiences, alongside Valencia-Sanchez et al.’s cautionary data, reaffirm that anti-thyroid antibody positivity is not an indication for empirical steroids (11). Optimal management requires reserving corticosteroid trials for patients with red-flag clinical features (especially the catatonia–delirium complex) and demonstrable CNS inflammatory markers. It also necessitates documenting outcomes with objective scales such as CGI-I, MoCA, BFCRS, and CAM to avoid misattributing nonspecific improvement to immunotherapy.

Significantly, we also identified anti-thyroid antibody-positive cases that exhibited a catatonic syndrome mimicking HE or had encephalopathy-like presentations but responded solely to ECT treatment, implying a spectrum of thyroid-related neuropsychiatric disorders ranging from incidental seropositivity and a potentially subthreshold autoimmune phenotype responsive to conventional psychotropics, and fully established steroid-responsive HE (5). These observations underscore the need to prioritize evidence-based psychiatric treatment before initiating immunotherapy, while reserving corticosteroids for patients meeting rigorous clinical and paraclinical criteria in the context of HE. This notion aligns with isolated case reports in the literature describing improvement of catatonia associated with anti-NMDA receptor encephalitis following ECT or memantine treatment.

Our study adds to the growing recognition that a subset of psychiatric inpatients with thyroid autoantibodies and atypical features—especially those with catatonia, delirium, and treatment resistance—may harbor undiagnosed autoimmune encephalopathy. Yet these patients are often overlooked in psychiatric settings, particularly when overt neurological findings are absent. Knowing that a small but important subgroup of patients may improve dramatically with steroids is crucial, especially as standard psychiatric treatments may be ineffective.

Few HE studies employ formal cognition metrics; in our nine confirmed cases, serial MoCA testing documented robust cognitive gains during corticosteroid therapy, often paralleling EEG and FDG-PET normalization. Earlier series (7, 32) relied largely on clinician impression, whereas combining MoCA with BFCRS in our cohort provides reproducible, objective evidence of treatment efficacy. Baseline MoCA scores were markedly low—likely reflecting the combined impact of catatonia and delirium, phenomena frequently under-recognized in HE literature. These findings highlight the need to integrate standardized neurocognitive assessments when monitoring autoimmune encephalopathy, especially in psychiatric settings where cognitive deficits are multifactorial. The study also illustrates the diagnostic overlap between HE and rapidly progressive neurodegenerative disorders, such as FTD or CJD, highlighted by two neurodegenerative cases whose catatonia resolved with electroconvulsive therapy.

Our findings carry several important implications for practice. They reinforce that HE should be considered in psychiatric inpatients with atypical or refractory presentations. Sudden-onset catatonia, unexplained delirium, or rapidly progressive cognitive decline – especially when accompanied by thyroid antibodies or other “red flag” signs – should prompt evaluation for autoimmune encephalopathy. Failure to consider HE may result in delayed diagnosis and inappropriate treatment, as standard psychiatric interventions alone may not address the underlying immune process.

Our study has limitations. It is a retrospective chart review. The number of confirmed HE cases was small (n=9) and all were women, potentially limiting generalizability and inflating the apparent frequency of thyroid autoimmunity. Effect sizes (e.g. 77.8% catatonia) should be interpreted cautiously until replicated in larger, mixed-gender cohorts. Requiring steroid responsiveness in the HE definition introduces circularity, possibly excluding cases that meet clinical criteria but were not treated or did not respond. Only 16 of 40 anti-thyroid antibody-positive patients underwent complete neurologic evaluation, so milder or subclinical cases may have been missed. It is also unclear whether the severe cognitive deficits observed were intrinsic to HE or secondary to catatonia/delirium. Given the high background prevalence of thyroid antibodies, their clinical significance even in this context remains uncertain. Most of our comparison group were anti-thyroid antibody positive psychiatric patients, which may exaggerate differences (spectrum bias). Elevated anti-TPO/TG antibodies may be epiphenomenal; T-cell–mediated inflammation, molecular mimicry, or yet-undiscovered neuronal antibodies could play primary roles (33). We did not employ advanced antibody discovery (rodent-brain assays), so novel autoantibodies may have been overlooked. Reliance on commercially available cell-surface antibody assays constitutes a methodological limitation, given the possibility of false-negative findings that may result in failure to detect underlying autoantibody related autoimmune processes (34).

Another limitation of this study lies in its dependence on chart reviews for historical psychiatric diagnoses and treatments, which were not regarded as causal evidence for Hashimoto’s encephalopathy (HE). The chronic and/or relapsing–remitting course of HE poses challenges in interpreting prolonged symptom histories, making it difficult to differentiate between the natural progression of HE and co-occurring primary psychiatric syndromes. Future studies with larger sample sizes are required to investigate the association between Hashimoto’s encephalopathy and preceding psychiatric disorders.

These limitations underscore the need for multicenter, prospective studies with standardized protocols, systematic catatonia/delirium screening, comprehensive CSF and imaging panels, and advanced immunologic testing. Such efforts are essential to refine HE prevalence, validate biomarkers, and optimize management in psychiatric populations.

## Conclusion and clinical implications

HE is an uncommon but highly treatable cause of refractory neuropsychiatric illness in psychiatric settings. A catatonia–delirium complex in the context of thyroid autoimmunity, negative routine neuronal antibodies, and failure of conventional psychotropics should prompt CSF analysis, metabolic imaging, and, when supportive, a carefully monitored corticosteroid trial. Early recognition can forestall prolonged disability and iatrogenic complications, whereas indiscriminate steroid use based solely on anti-thyroid antibody positivity risks harm. Multimodal, interdisciplinary assessment—including systematic BFCRS, CAM, MoCA, and paraclinical testing—offers the best balance between timely treatment and diagnostic precision. Future multicenter, prospective studies with advanced immunological assays are required to delineate true HE prevalence, clarify pathogenesis, and optimize long-term immunotherapy strategies.

## Data Availability

The original contributions presented in the study are included in the article/Supplementary Material. Further inquiries can be directed to the corresponding authors.

## References

[1] Brain L Jellinek EH Ball K. Hashimoto’s disease and encephalopathy. Lancet. (1966) 2:512–4. doi: 10.1016/S0140-6736(66)92876-5, PMID: 4161638

[2] Chong JY Rowland LP Utiger RD. Hashimoto encephalopathy: syndrome or myth? Arch Neurol. (2003) 60:164–71. doi: 10.1001/archneur.60.2.164, PMID: 12580699

[3] Laurent C Capron J Quillerou B Thomas G Alamowitch S Mekinian A. Steroid-responsive encephalopathy associated with autoimmune thyroiditis (SREAT): characteristics, treatment and outcome in 251 cases from the literature. Autoimmun Rev. (2016) 15:1129–33. doi: 10.1016/j.autrev.2016.09.008, PMID: 27639840

[4] Endres D Perlov E Riering AN Maier V Stich O Dersch R. Steroid-responsive chronic schizophreniform syndrome in the context of mildly increased antithyroid peroxidase antibodies. Front Psychiatry. (2017) 8:64. doi: 10.3389/fpsyt.2017.00064, PMID: 28484400 PMC5399039

[5] Mattozzi S Sabater L Escudero D Ariño H Armangue T Simabukuro M. Hashimoto encephalopathy in the 21st century. Neurology. (2020) 94:e217–24. doi: 10.1212/WNL.0000000000008785, PMID: 31882532

[6] Ferracci F Bertiato G Moretto G. Hashimoto’s encephalopathy: epidemiologic data and pathogenetic considerations. J Neurol Sci. (2004) 217:165–8. doi: 10.1016/j.jns.2003.09.007, PMID: 14706219

[7] Castillo P Woodruff B Caselli R Vernino S Lucchinetti C Swanson J. Steroid-responsive encephalopathy associated with autoimmune thyroiditis. Arch Neurol. (2006) 63:197–202. doi: 10.1001/archneur.63.2.197, PMID: 16476807

[8] Graus F Titulaer MJ Balu R Benseler S Bien CG Celluci T. A clinical approach to diagnosis of autoimmune encephalitis. Lancet Neurol. (2016) 15:391–404. doi: 10.1016/S1474-4422(15)00401-9, PMID: 26906964 PMC5066574

[9] Pollak TA Lennox BR Müller S Benros EM Prüss H van Elst LT. Autoimmune psychosis: an international consensus on an approach to the diagnosis and management of psychosis of suspected autoimmune origin. Lancet Psychiatry. (2020) 7:93–108. doi: 10.1016/S2215-0366(19)30290-1, PMID: 31669058

[10] Dubey D Kothapalli N McKeon A Flanagan EP Lennon VA Klein CJ. Predictors of neural-specific autoantibodies and immunotherapy response in patients with cognitive dysfunction. J Neuroimmunol. (2018) 323:62–72. doi: 10.1016/j.jneuroim.2018.07.009, PMID: 30196836

[11] Cummings JL Mega M Gray K Rosenberg-Thompson S Carusi DA Gornbein J. The Neuropsychiatric Inventory: comprehensive assessment of psychopathology in dementia. Neurology. (1994) 44:2308–14. doi: 10.1212/WNL.44.12.2308, PMID: 7991117

[12] Bush G Fink M Petrides G Dowling F Francis A Catatonia. I. Rating scale and standardized examination. Acta Psychiatr Scand. (1996) 93:129–36. doi: 10.1111/j.1600-0447.1996.tb09814.x, PMID: 8686483

[13] Inouye SK van Dyck CH Alessi CA Balkin S Siegal AP Horwithz RI. Clarifying confusion: the confusion assessment method. A new method for detection of delirium. Ann Intern Med. (1990) 113:941–8. doi: 10.7326/0003-4819-113-12-941, PMID: 2240918

[14] Nasreddine ZS Phillips NA Bédirian V Charbonneau S Whitehead V Collin I. The Montreal Cognitive Assessment, MoCA: a brief screening tool for mild cognitive impairment. J Am Geriatr Soc. (2005) 53:695–9. doi: 10.1111/j.1532-5415.2005.53221.x, PMID: 15817019

[15] American Psychiatric Association. Diagnostic and statistical manual of mental disorders. 4th ed. Washington, DC: American Psychiatric Association (1994).

[16] Guy W. ECDEU assessment manual for psychopharmacology. Rockville, MD: US Department of Health, Education, and Welfare (1976).

[17] Figgie MP Jr Kelly H Pyatka N Chu C Abboud H. Characterization of neurological morbidity associated with thyroid antibodies: Hashimoto’s encephalopathy and beyond. J Neurol Sci. (2024) 458:122908. doi: 10.1016/j.jns.2024.122908, PMID: 38309249

[18] Dumrikarnlert C Thakolwiboon S Senanarong V. Clinical presentations and treatment outcomes of Hashimoto encephalopathy at Siriraj Hospital–Thailand’s largest national tertiary referral center. BMC Neurol. (2023) 23:334. doi: 10.1186/s12883-023-03305-4, PMID: 37737161 PMC10514970

[19] Valencia-Sanchez C Pittock SJ Mead-Harvey C Dubey D Flanagan EP Lopez-Chiriboga S. Brain dysfunction and thyroid antibodies: autoimmune diagnosis and misdiagnosis. Brain Commun. (2021) 3:fcaa233. doi: 10.1093/braincomms/fcaa233, PMID: 34061124 PMC8152924

[20] Tsai CH Yu KT Chan HY Chan CH. Hashimoto’s encephalopathy presenting as catatonia in a bipolar patient: a case report. Asian J Psychiatr. (2021) 66:102895. doi: 10.1016/j.ajp.2021.102895, PMID: 34741883

[21] Chen YW Hung PL Wu CK Tseng PT. Severe complication of catatonia in a young patient with Hashimoto’s encephalopathy comorbid with Cornelia de Lange syndrome. Kaohsiung J Med Sci. (2015) 31:60–1. doi: 10.1016/j.kjms.2014.06.001, PMID: 25600923 PMC11916868

[22] Johnson ET Eraly SG Subramaniyam BA Muliyala KP Moirangthem S Reddi VSK. Complexities of co-occurrence of catatonia and autoimmune thyroiditis in bipolar disorder: a case series and selective review. Brain Behav Immun Health. (2022) 22:100440. doi: 10.1016/j.bbih.2022.100440, PMID: 36118271 PMC9475125

[23] Bharadwaj B Sugaparaneetharan A Rajkumar RP. Graves’ disease presenting with catatonia: a probable case of encephalopathy associated with autoimmune thyroid disease. Acta Neuropsychiatr. (2012) 24:374–9. doi: 10.1111/j.1601-5215.2012.00654.x, PMID: 25287181

[24] Lee Y House EM. Treatment of steroid-resistant Hashimoto encephalopathy with misidentification delusions and catatonia. Psychosomatics. (2017) 58:322–7. doi: 10.1016/j.psym.2016.10.008, PMID: 28190544

[25] Ali HT Alnuaimi A Alshehhi A Alshamsi AM Al Awadhi A. Catatonia as the presentation of encephalopathy associated with autoimmune thyroiditis: a case report and literature review. J Psychiatr Pract. (2023) 29:499–504. doi: 10.1097/PRA.0000000000000751, PMID: 37948176

[26] Sarkis RA Coffey MJ Cooper JJ Hassan I Lennox BR. Anti-N-Methyl-D-Aspartate Receptor Encephalitis: a review of psychiatric phenotypes and management considerations. J Neuropsychiatry Clin Neurosci. (2019) 31:137–42. doi: 10.1176/appi.neuropsych.18010005, PMID: 30561283

[27] Chaudhuri J Mukherjee A Chakravarty A. Hashimoto’s encephalopathy: case series and literature review. Curr Neurol Neurosci Rep. (2023) 23:167–75. doi: 10.1007/s11910-023-01255-5, PMID: 36853554 PMC9972331

[28] Endres D Runge K Meixensberger S Feige B Denzel D Pankratz B. An observational study on the association of anti-thyroid autoantibodies with clinical, EEG, MRI, FDG-PET, cerebrospinal fluid and anti-neuronal antibody findings in 530 patients with schizophreniform and affective disorders. Psychoneuroendocrinology. (2021) 131:105320. doi: 10.1016/j.psyneuen.2021.105320, PMID: 34171794

[29] Ferracci F Carnevale A. The neurological disorder associated with thyroid autoimmunity. J Neurol. (2006) 253:975–84. doi: 10.1007/s00415-006-0170-7, PMID: 16786216

[30] Menon V Subramanian K Thamizh JS. Psychiatric presentations heralding Hashimoto’s encephalopathy: a systematic review and analysis. J Neurosci Rural Pract. (2017) 8:261–7. doi: 10.4103/jnrp.jnrp_440_16, PMID: 28479803 PMC5402495

[31] Olmez I Moses H Sriram S Kirshner H Lagrange AH Pawate S. Diagnostic and therapeutic aspects of Hashimoto’s encephalopathy. J Neurol Sci. (2013) 331:67–71. doi: 10.1016/j.jns.2013.05.009, PMID: 23759502

[32] Litmeier S Prüss H Witsch E Witsch J. Initial serum thyroid peroxidase antibodies and long-term outcomes in steroid-responsive encephalopathy associated with autoimmune thyroiditis. Acta Neurol Scand. (2016) 134:452–7. doi: 10.1111/ane.12556, PMID: 26757046

[33] Tyler KL Rüegg S. The neuromythology of Hashimoto encephalopathy: the emperor has no clothes. Neurology. (2020) 94:55–6. doi: 10.1212/WNL.0000000000008776, PMID: 31882524

[34] Dalmau J Graus F. Diagnostic criteria for autoimmune encephalitis: utility and pitfalls for antibody-negative disease. Lancet Neurol. (2023) 22:529–40. doi: 10.1016/S1474-4422(23)00083-2, PMID: 37210100

